# The Reproach of Nurses' Poor Pay

**Published:** 1919-02-08

**Authors:** 


### NEW MINISTRY OF HEALTH PROPOSALS.

### The Reproach of Nurses' Poor Pay.

**"A great development of maternity and nursing services is wanted. I seize the present opportunity to say something on this particular subject. I believe the provision of skilled and well-trained midwives and nurses, properly paid, is an essentially vital part of our health policy. And, I say, you will never enjoy an adequate supply of well-trained experienced nurses so long as you are content to pay them the wages you pay to a scullery-maid."**

This was the message to nurses which Dr. Addison, President of the Local Government Board, delivered with particular emphasis in the course of his speech at Kingsway Hall, London, on Monday night with reference to the future health activities of his department. The hall was well filled, and the platform was representative of the various interests directly affected by the proposed Ministry of Health.

### The Slaughter of the Innocents.

Sir Kingsley Wood, M.P., who presided, said he had received a message from the Prime Minister expressing his sympathy with the effort to secure a drastic reform of health administration in the near future. Many hundreds of members of Parliament, the chairman continued, were definitely committed to the incorporation of a Health Ministry, and thousands of health workers were determined that the delay which had dogged its footsteps should be ended. At least a quarter of a million children had died since the outbreak of the war whose lives might have been saved, and there were in our schools upward of a million weak and defective children. Consumption was as widespread as was smallpox in the 18th century, disabling 150,000 persons every year. They did not desire the new Ministry to be permanently associated with the Poor Law. (Hear, hear.) Money spent for medical research was the best insurance premium the nation could pay against death and destitution. Prevention should be the great watchword of the proposed New Ministry.

### Too Many "Cooks."

Dr. Addison said he accepted this measure as a legacy from a man (the late Lord Rhondda) who had worn himself to death in the interests of his country, and he took his present office on the understanding that this Bill would be passed through Parliament as rapidly as possible. At present, doing odds and ends of health work, were no fewer than twenty-one Government departments and more than 2,000 local authorities; he intended to open the eyes of the public by publishing a White Paper on the subject. In these circumstances could they be surprised that progress in health matters was deadly slow? In working a Health Ministry the good will and help would be needed of local authorities, insurance committees, as well as the medical, nursing, and midwifery services. Large units would be needed for administration, they must lift up their eyes above the parish pump and take big views. (Hear, hear.) He believed in an Advisory Council, and before the bill passed he intended to form a body of persons, experienced and capable, to give advice and pass criticism on local Government, medical, insurance, scientific, and health matters generally. Public opinion must make its influence felt in the modelling of the policy; the help of all outside agencies was needed. (Dr. Addison then made his reference to nurses given at the head of this report.) Then facilities for diagnosis must be organised; medical men in the Army had discovered what team work could do in scientific medicine, and something of the same kind was needed for home work.

### Hospital Building Should Not Cease.

People who proposed to build hospitals had asked him whether they should stay their hand. They need not be alarmed; every branch of the health services would have to be made use of. The traditions of the medical profession or of the British public were not going to be uprooted in that direction. Diagnosis and early treatment, laboratory and clinical facilities, and nursing provision were needed throughout the land, and it was possible to provide these without interfering with the susceptibilities or proper ambitions of any one. (Applause.) Prevention was the keynote, and we should have to short-circuit a lot of our old methods, especially on housing, the position in regard to which was most grave. Authority would be asked for to deal in a common sense way with restrictive by-laws, and it was not proposed to proceed by compensation.

### A Council of Wives.

Viscountess Rhondda, speaking in support, urged Dr. Addison to make full use of women. Besides the expert Advisory Council, she would like to see a Council of ordinary wives and mothers acting as a link between the Ministry and the people's homes. (Applause.)

### Medical Men's Doubts.

Sir Bertrand Dawson, Physician-in-Ordinary to the King, said the attitude of the medical profession towards this Ministry of Health was one of warm-hearted sympathy and earnest support. At the same time there was watchfulness tinged with doubt—doubt which he thought would be dissolved by the remarks of Dr. Addison that night. The medical man led an extremely hard life, after years of preparation and expense, and it was no wonder he hesitated before accepting any scheme that vitally affected him. In any national plan the medical man looked for better conditions for carrying on his work, and he (Sir Bertrand) thought he would get them from this Ministry. Medical men who had worked at the front had learned to do things under proper hospital conditions, and they dreaded coming back to "the surgery, the bottle of medicine, and the ill-paid bill." Again, the medical man at present was too much under the control of lay people; he wanted more freedom. For instance, the status of the Insurance Committees would have to be modified. (Applause.) As to the public aspect, in the future no community must be considered complete without its fully equipped hospital. There must be open spaces for games and recreation conducted in an organised manner, with the majority participating and not merely looking on as at present. With physical culture of this sort he believed much of the irritability and unrest of the times would disappear. (Hear, hear.)

### The Stigma of the Poor Law.

Sir Alfred Warren, M.P., Parliamentary Agent, Manchester Unity of Oddfellows, proposed a resolution calling upon the Government to give early facilities for the immediate establishment of a Ministry of Health, and to incorporate in the Bill a definite declaration that the non-medical side of the Poor Law and other health functions shall be dissociated at an early date from the Health Ministry. He said the diverse interests consulted by Dr. Addison had resolved to give the Minister whole-hearted support towards bringing the scheme into existence early, but the stigma of the Poor Law must be removed. He was glad there was to be no compulsion; the country was sick to death of compulsion. (Applause.)

Mr. W. A. Appleton (secretary General Federation of Trades Unions) seconded, and his advice was "Do it now." The nation wanted something at once. He strongly advised that the "ordinary" woman should be placed on the Advisory Council.

Mr. A. C. Thompson (Prudential Approved Societies) and Mr. P. Rockliffe (Joint Committee of Approved Societies) supported the resolution, which was adopted.

Dr. Addison, in reply to questions, said he wished to make it clear that they intended to dissociate the Poor Law system generally from their health measures. The service of women generally would also be sought.

